# Effect of multimodality chest physiotherapy in prevention of ventilator-associated pneumonia: A randomized clinical trial

**DOI:** 10.4103/0972-5229.68218

**Published:** 2010

**Authors:** Renu B. Pattanshetty, G. S. Gaude

**Affiliations:** **From:** KLE Institute of Physiotherapy, Belgaum 590010, India

**Keywords:** Clinical Pulmonary infection Score, multimodality chest physiotherapy, ventilator-associated pneumonia

## Abstract

**Background:**

Despite remarkable progress that has been achieved in the recent years in the diagnosis, prevention, and therapy for ventilator-associated pneumonia (VAP), this disease continues to create complication during the course of treatment in a significant proportion of patients while receiving mechanical ventilation.

**Objective:**

This study was designed to evaluate the effect of multimodality chest physiotherapy in intubated and mechanically ventilated patients undergoing treatment in the intensive care units (ICUs) for prevention of VAP.

**Patients and Methods:**

A total of 101 adult intubated and mechanically ventilated patients were included in this study. Manual hyperinflation (MH) and suctioning were administered to patients in the control group (*n* = 51), and positioning and chest wall vibrations in addition to MH plus suctioning (multimodality chest physiotherapy) were administered to patients in the study group (*n* = 50) till they were extubated. Both the groups were subjected to treatment twice a day. Standard care in the form of routine nursing care, pharmacological therapy, inhalation therapy, as advised by the concerned physician/surgeon was strictly implemented throughout the intervention period.

**Results:**

Data were analyzed using SPSS window version 9.0. The Clinical Pulmonary infection Score (CPIS) Score showed significant decrease at the end of extubation/successful outcome or discharge in both the groups (*P* = 0.00). In addition, significant decrease in mortality rate was noted in the study group (24%) as compared to the control group (49%) (*P* = 0.007).

**Conclusions:**

It was observed in this study that twice-daily multimodality chest physiotherapy was associated with a significant decrease in the CPIS Scores in the study group as compared to the control group suggesting a decrease in the occurrence of VAP. There was also a significant reduction in the mortality rates with the use of multimodality chest physiotherapy in mechanically ventilated patients.

## Introduction

Pneumonia is the single most common nosocomial infection among patients undergoing treatment in the intensive care units (ICUs). Despite considerable progress that has been achieved in the recent past in the diagnosis, prevention, and therapy of hospital-acquired infections, ventilator-associated pneumonia (VAP) continues to create complications during the course of treatment in the case of a significant proportion of patients receiving mechanical ventilation. Mortality rates among patients with VAP have been reported to be increasing at alarming proportions to register a mortality rate of as high as 72%, and the morbidity associated with VAP is also observed to be considerably contributing to the increase in the number of days of hospital stay as well as to the enormous increase in the healthcare costs.[1]

Many risk factors have been associated with VAP, which include among others the presence of chronic obstructive pulmonary disease (COPD), airway intubation, reduced conscious state, intracranial monitoring, airway reintubation, mechanical ventilation greater than 7 days, use of positive end expiratory pressure (PEEP) and supine patient positioning. Airway intubation and mechanical ventilation, in particular, reduce the normal clearance of airway secretions, increasing the risk of VAP. There is evidence showing that aggressive preventive measures may reduce the high rates of morbidity associated with VAP in patients who are critically ill.[2] There is also supportive evidence confirming that various combinations of chest physiotherapy have played their respective significant roles to assist in the re-expansion of the lung and confer short-term improvement in total lung-thorax compliance and expiratory flow rates.[3] However, there is hardly any evidence so far to show that multimodality chest physiotherapy assists in the prevention or treatment of VAP.

This study prospectively investigated the effect of multimodality chest physiotherapy in intubated and mechanically ventilated patients in the ICUs on the prevalence of VAP. Furthermore, secondary outcome of interest, such as the effects of multimodality chest physiotherapy on the duration of mechanical ventilation and the number of days of physical stay in the ICUs, were also included in this study.

## Patients and Methods

The study was undertaken in a tertiary care referral hospital over a period of one year. All adult patients who had undergone mechanical ventilation for >48 h from the ICUs, referred by the concerned physician, surgeon, or the intensivist were recruited into the study. All those patients who suffered from acute respiratory distress syndrome, acute pulmonary edema, untreated pneumothorax, those requiring high-respiratory support with FiO_2_ > 0.70, acute myocardial infarction, cardiac arrhythmias, hypovolemia, hemodialysis, intubation <48 h, community-acquired pneumonia, unstable cardiovascular or neurological function or injury preventing positioning for chest physiotherapy, open heart surgeries, admission with tracheostomy, and HIV-positive patients were excluded from the study.

Ethical approval was obtained from the Research Committee of the KLE University before the commencement of the study. All the patients who were enrolled in this study were ventilated by Servo Ventilator-900C and Servo Ventilator 300. After obtaining written consent from the patients or the patients’ relatives, baseline data including age, gender, admission diagnosis, ventilatory mode, radiological features suggesting pneumonia, Glasgow Coma Score, and CPIS Score of all patients were noted.[4] After obtaining the baseline data, the patients were randomly allocated to one of the two groups, i.e., the control group or the study group by envelope method. Manual hyperinflation (MH) and suctioning were administered to the patients in the control group while patients in the study group received positioning and chest wall vibrations in addition to MH plus suctioning.

Standard care in the form of routine nursing care, pharmacological therapy, bronchodilators, antibiotics inhalation therapy, as advised by the concerned physician, surgeon, or the intensivist was strictly implemented throughout the intervention. The management of the patient was left entirely at the discretion of the treating physician/surgeon. The changes in ventilatory parameters were adjusted by the intensivist according to the condition of the patient. All patients in both groups were treated with chest physiotherapy twice a day (9.30 a.m. and 3.30 p.m.) till they were weaned off from the ventilator [Table 5] and were followed up for the global outcome in terms of recovery, death, total length of stay on ventilation, discharge against medical advice, or any other complications.

**Table 5:** Weaning criteria[31]

Ventilator criteria: Spontaneous Vt > 5– 8 mL/kg Spontaneous RR (f) < 30/min PaO_2_ < 50 mmHg with normal pH
Oxygenation criteria: PaO_2_ with PEEP >100 mmHg at FiO_2_ up to 0.4. SaO_2_ < 90% 2FiO_2_ up to 0.4 PaO_2_/FiO_2_ > 200 mmHg PaO_2_ > 60 mmHg RSBI < 80

The following modes of chest physiotherapy maneuvers were used for the patients under mechanical ventilation.

### Manual hyperinflation

In order to ensure a uniform and correct technique, MH was employed by the principal investigator to all the patients. A 2.0-L reusable manual resuscitator (Hudson RCI-nondisposable and autoclavable (silicone) was used to deliver the MH breaths. It was connected to a flow of 100% oxygen at 15 L/min. The resuscitator was slowly compressed with both hands, and an inspiratory breath was maintained for 3–5 s at the end of half of the resuscitator and then it was kept completely pressed. The resuscitator expiration was maintained at passive mode and unobstructed to facilitate expiratory flow with no positive end expiratory pressure applied. Sufficient time was allowed for the resuscitator to fill completely prior to the next breath. The MH procedure was carried out daily at the rate of 8–13 breaths/min for a period of 20 min at each session twice a day (9.30 a.m. and 3.30 p.m.). After MH, immediately chest vibrations were also employed.

### Chest vibrations

Chest vibration[6] defined as the manual application of a fine oscillatory movement combined with compression to the patient’s chest wall which helps to loosen and mobilize the secretions was given prior to suctioning. The patient was positioned in supine, and then randomly positioned either to right or left side lying in the bed. The principal investigator placed her hands anteriorly and laterally on the patient’s chest with fingers placed in the inter-rib space, and then applied vibrations in the expiratory phase of breathing. This technique was repeated thrice in each of the three zones, i.e., upper zone, middle zone, and lower zone of the chest.

### Suctioning

Duration of endotracheal suctioning[7] was limited to 15 s. To standardize the suctioning procedure and to enhance aspiration of dry secretions in some patients, 1 mL of normal saline via the tracheal tube before MH was done. The suctioning session involved instillation of 1 mL of normal saline in the tracheal tube, followed by suctioning once every minute for 4 min. The sizes of the suctioning catheters used were FG14, and FG16 (Romson’s 53 cm in length). The suctioning device was inserted fully up to the carina and then withdrawn for 1 cm prior to the application of negative pressure. During suctioning, the specimen from the lower respiratory tract was collected in a sterile container after instillation of 1 mL of normal saline for culture and sensitivity testing.

### Positioning

At the end of the treatment session, i.e., after suctioning, the head end was arranged to be positioned at an angle of elevation in the range of 30-45° (as measured by a protractor) and this positioning was maintained for minimum of 30 min for improving the ventilation in all patients.[8] The change of positioning from lying supine to lateral orientations by turning the patients was manually done by nursing staff once in every 2 h. The MH and vibrations were administered by the principal investigator who also carries the responsibility to ensure the uniformity in the technique. The suctioning and positioning operations for the patients were assisted by the trained intern/trained nursing staff.

### Statistical analysis

Data were analyzed using SPSS window version 9.0. Demographic data were analyzed using Chi-Square test, which included gender, admission to various ICUs, and global outcome in the form of death/mortality, successful outcome, or discharge against medical advice. Mann–Whitney U-tests were used to analyze the number of days the patients spent under ventilation, and the number of days of the patient’s stay in the ICU. Students paired and unpaired ‘t’-tests were used to compare the results between and within the groups, respectively.

## Results

A total of 101 adult patients who had undergone intubation and kept under mechanical ventilation were enrolled as the subjects for this study. Of 101 patients, 51 patients were allocated to the control group and 50 patients to the study group. The two trial groups were well matched for age, sex, baseline CPIS Scores, Glasgow Coma Scores (*P* = 0.1) initial ventilatory modes, and admission to various ICUs. There was no statistical difference with respect to VAP ≥ 5 days or VAP ≤ 5 days between both the groups. Neither the number of days of ventilation nor the number of days’ stay in the ICU showed any statistical significance (*P* = 0.986 and *P* = 0.102, respectively) between both groups [Table 1]

**Table 1:** Baseline data of both the groups

Variables	Control group (*n* = 51)	Study group (*n* = 50)	“t” Value
Age	51.6 ± 17.47	47.8 ± 14.72	1.172
Males	40	37	0.274
Females	11	13	
CPIS at the initiation of physiotherapy	7.6 ± 1.75	7.6 ± 1.15	0.108
VAP ≥ 5 days	45	46	
VAP ≤ 5 days	2	2	
Ventilatory mode			
SIMV + PS	37	35	
VC	13	15	
PS	1	0	
Type of ICU stay			
Neurosurgery ICU	9	8	1.762
Medical ICU	30	26	
Surgical ICU	7	12	
Cardiac ICU	5	04	

CPIS Score, the main outcome variable, was statistically significant at the end of extubation/successful outcome or discharge as compared to baseline CPIS score in each group [Table 3]. Mortality rate was higher in the control group (49%) than that in the study group (24%) which was statistically significant (*P* = 0.007) [Table 1]. Weaning of ventilation was successful in the case of 62% of the patients in the study group as compared to 31.37% of the patients in the control group which was statistically significant (*P* = 0.007). Mean reduction in the CPIS Scores between both the groups was highly significant (*P* = 0.000) as compared to the baseline CPIS Score [Table 3]. The CPIS Score reduction was more prominent in the study group (3.9 ± 1.69) with significant reduction (*P* = 0.000) when compared with the baseline data in both the groups, and the reduction in CPIS Score was higher in the study group (3.4 ± 4.4) as compared to the control group (1.9 ± 2.9) [Tables 2 and 4]

**Table 2:** Final outcome variables of both the groups

Variables	Control group (*n* = 51)	Study group (*n* = 50)	“t” Value
CPIS score at end of intubation /discharge from ICU	5.2 ± 2.17	3.7 ± 1.43	4.077
Number of days of intubation	8.5 ± 5.21	8.7 ± 5.60	0.196
Number of days of ICU stay	11.3 ± 5.73	13.9 ± 9.77	1.651
Deaths	25 (49.09%)	12 (24%)	
Successful weaning	16 (31.37%)	31 (62%)	
AMA	10 (19.60%)	7 (14%)	

**Table 3:** Mean reduction in CPIS scores between 2 groups (a) before and (b) after chest physiotherapy

	CPIS score	*P* value
(a) Before chest physiotherapy		
Control group	2.4 ± 1.62	0.000
Study group	3.9 ± 1.69	
(b) After chest physiotherapy		
Control group	10.586 (1.9 ± 2.9)	0.000
Study group	16.434 (3.4 ± 4.4)	0.000

**Table 4:** CPIS score in relation to the outcome

	CPIS score	*P* value
Successful weaning	3.3 ± 1.16	0.00
Death	5.4± 2.10	0.00
Discharge against medical advice	5.5 ± 1.91	0.00

## Discussion

The critically ill patients in this study were intubated and mechanically ventilated for at least 48 h. All patients in the control group received chest physiotherapy in form of MH and suctioning; those in the study group, however, received the multimodality chest physiotherapy (MH, suctioning, chest vibrations, and patient positioning). CPIS Score, the main outcome variable, was statistically significant at the end of extubation/successful outcome or discharge in both the groups.

This study revealed that mortality rate was observed to be higher in the control group (49%) than that in the study group (24%) which was statistically significant (*P* = 0.007). Successful weaning was observed in 62% of the patients in the study group as compared to 31.37% of patients in the control group which was statistically significant (*P* = 0.007). Mean reduction in the CPIS Scores between both the groups was highly significant (*P* = 0.000) as compared to the baseline CPIS. Score’s reduction was higher in study group (3.4 ± 4.4) as compared to that in the control group (1.9 ± 2.9) (*P* < 0.000).

Ventilator-associated pneumonia is defined as parenchymal lung infection occurring more than 48 h after initiation of mechanical ventilation. The segregation of patients with VAP into groups categorizing them as early- and late-onset has been shown to be of paramount importance. Early-onset pneumonia, i.e., VAP ≤ 5 days commonly results from aspiration of endogenous community-acquired pathogens such as *Staphylococcus aureus, Streptococcus pneumoniae*, and *Haemophilus influenzae* with endotracheal intubation and impaired consciousness being the associated main risk factors. Late-onset pneumonia, i.e., VAP ≥ 5 days followed by aspiration of oropharyngeal or gastric secretions containing potentially drug-resistant nosocomial pathogens. Is associated with an attributable excess mortality. The immediate administration of treatment is crucial in VAP, and inappropriate treatment is associated with an increased risk of death due to pneumonia.[9] In addition to antimicrobial treatment, several risk factors for VAP can be minimized by simple and inexpensive preventive strategies. Chest physiotherapy is one such common preventive strategy where chest physiotherapists routinely treat most of the ICU patients with various chest physiotherapy techniques such as MH, suctioning, patient positioning, chest vibrations, chest percussions, various coughing techniques in combination or individually to prevent pulmonary complications like VAP and atelectasis in the ICUs.[10] Effects on arterial oxygenation, hemodynamic ventilatory effects, and changes in total lung/thorax compliance following chest physiotherapy have been well documented in various previous studies.[11–13] Though, many studies have investigated the short-term effects of multimodality respiratory physiotherapy on pulmonary functions of the intubated ICU patients receiving mechanical ventilation, there have been very few studies to study the effects of multimodality respiratory physiotherapy in prevention of the VAP and decrease in the mortality rates in the case of patients in ICUs.[14] Intubation and mechanical ventilation may impair mucociliary clearance and lead to sputum retention, airway occlusion, atelectasis, and VAP. Mucociliary clearance depends on the complex interaction between ciliated columnar epithelial cells of tracheobronchial tree and the special viscoelastic properties of the bronchial secretions. The mucociliary system represents an important protective mechanism of the upper and lower respiratory tract whereby inhaled particles and microorganisms are removed from the tracheobronchial system. Patients in the ICU have a tendency to retain secretions particularly while receiving long-term mechanical ventilation and, further, they are at the highest risk of contracting pneumonia.[15] Chest physiotherapy has an important role as part of the intensive care team’s care to optimally manage the clearance of the tracheobronchial airway.[16]

Manual hyperinflation is one of the airway clearance techniques routinely employed by physiotherapists in critical care settings, especially in the management of patients subjected to intubation and mechanical ventilation. Effects of MH on various respiratory parameters of the patients under intubation and mechanical ventilation have been well documented by Paratz *et al*._*L*_[17] which have shown to significantly increase lung compliance (C_L_) and PaO_2_: FiO_2_, and decrease (A-a)PO_2_. In another study, Mervyn *et al*.[18] have observed hemodynamic changes in heart rate, blood pressure, and cardiac output following MH, and these may be related to tidal volume although other factors such as generation of high level of intrinsic PEEP may also be attributed to such changes.

Paratz *et al*.[17] have shown MH as a technique which has resulted in improvement in gas exchange in patients with lung disease due to extrapulmonary events and did not result in impairment of the cardiovascular system. Jessica *et al*.[19] have demonstrated that MH in conjunction with suctioning has demonstrated beneficial changes in respiratory mechanics in patients receiving mechanical ventilation with VAP, although they did not study the effects of MH plus suction on the prevention and treatment of VAP.

Effective suctioning is an essential aspect of airway management and has an important role to play in the prevention of VAP, especially early-onset VAP.[20] In this study, the control group received MH plus suctioning with open suctioning system. Seymour *et al*. have effectively studied the physiologic impact of closed system endotracheal suctioning in spontaneously breathing patients receiving mechanical ventilation, and they observed significant and sustained alterations in cardiac variables, respiratory pattern and lung volumes after using closed suctioning system method. However, the clinical importance of such findings remains unknown.[21] Endotracheal suctioning may also have side effects or complications which may lead to partial lung collapse which may further lead to desaturation. Almgren *et al*. investigated the effects of endotracheal suctioning in volume-controlled ventilation (VCV) and pressure-controlled ventilation (PCV) with open suctioning system (OSS) or closed suctioning system (CSS), and they observed that PCV caused more lung collapse leading to impaired gas exchange which was more severe and persistent than in VCV.[22] In this study, 38 patients were initiated with synchronized intermittent mandatory ventilation (SIMV) with PS and 13 patients with volume-control mode in the control group as against 35 patients with SIMV with PS and 15 patients with volume-control mode in the study group. Mortality rate was decreased in the study group as compared to the control group. However, correlation between the different modes of ventilation and mortality was beyond the scope of this study.

Vibration is a manual technique used widely to assist the removal of pulmonary secretions.[6] Kim *et al*. has explained the theoretical effects of vibrations on secretion clearance. The expiratory flow rates generated during vibrations do not adequately augment secretion clearance by annular flow. *In vitro* studies suggest that annular flow can assist removal of secretions when there is an expiratory bias to airflow which results in a mass movement of secretions by annular flow toward the mouth if critical volume and thickness of secretions are present.[23] Effectiveness of vibrations has been evaluated in various studies.[24,25] Eales *et al*. investigating 37 patients receiving mechanical ventilation after cardiac surgery found that arterial blood gas analyses (ABG) values and lung compliance did not significantly change during the treatment course with MH and suction with or without the vibrations. However, effect of vibrations in prevention of VAP needs to be established.[26]

One of the simplest and the least expensive measures in preventing VAP is maintaining the patient’s head end of the bed in an elevated position. Increasing the angle of the head end of the bed is effective because it decreases the risk of aspiration of both gastric contents and secretions from the upper aerodigestive tract. These secretions are often colonized with potentially pathogenic bacteria, and generally colonization precedes infection.[26] Hess has proposed various procedures such as use of rotational beds, prone position, and semi-recumbent position as procedures to prevent VAP.[27] The use of semi-recumbent positioning has also been addressed in evidence-based guidelines.[28] In the absence of medical contraindications, elevation of the head end of the bed of patients at an angle of 30–45° may help in decreasing the risk of VAP.[29]

Ntoumenopoulos *et al*.[14] studied the effects of multimodality chest physiotherapy (gravity-assisted drainage or positioning, chest vibrations, and suctioning), in 60 patients receiving mechanical ventilation and concluded that chest physiotherapy in the ventilated patients was independently associated with a reduction in VAP. The use of chest physical therapy for patients with a variety of pulmonary problems is a well entrenched practice in standard medical care. The evidence in support of these techniques is inconsistent and variable, and the literature on this account seems to abound with confusion and conflicts. The clinical effectiveness of chest physiotherapy for pneumonia is controversial. Some clinical studies have concluded that chest physiotherapy does not hasten the resolution of pneumonia.[30] However, there has been a lack of systematic review or meta-analysis of chest physiotherapy for pneumonia, and no study has been published yet.[14]

*In conclusion*, this study of critically ill patients who were subjected to intubation and receiving mechanical ventilation demonstrated that twice-daily multimodality chest physiotherapy in the form of MH, endotracheal suctioning (OSS), chest wall vibrations, and semi-recumbent patient positioning to 30–45° of elevation was associated with decrease in CPIS scores in the study group as compared to the control group suggesting a decrease in the occurrence of VAP and mortality rate. Further study in the form of larger randomized controlled trials using combination of various chest physiotherapy techniques is suggested to confirm these findings which hold the most therapeutic potential in the prevention of VAP.
